# Age-dependent inverse correlations in CSF and plasma amyloid-β(1–42) concentrations prior to amyloid plaque deposition in the brain of 3xTg-AD mice

**DOI:** 10.1038/srep20185

**Published:** 2016-02-02

**Authors:** Soo Min Cho, Sejin Lee, Seung-Hoon Yang, Hye Yun Kim, Michael Jisoo Lee, Hyunjin Vincent Kim, Jiyoon Kim, Seungyeop Baek, Jin Yun, Dohee Kim, Yun Kyung Kim, Yakdol Cho, Jiwan Woo, Tae Song Kim, YoungSoo Kim

**Affiliations:** 1 Center for Neuro-Medicine, Hwarangno 14-gil 5, Seongbuk-gu, Seoul, Republic of Korea; 2 Biological Chemistry Program, Korea University of Science and Technology, 217 Gajungro, Yuseong-gu, Daejeon, Republic of Korea; 3 GoshenBiotech, 83-2 Wolmun-ri, Wabu-eup, Namyangju-si, Gyeonggi-do, Republic of Korea; 4 Center for Neuroscience and Hwarangno 14-gil 5, Seongbuk-gu, Seoul, Republic of Korea; 5 Center for BioMicroSystems, Brain Science Institute, Korea Institute of Science and Technology, Hwarangno 14-gil 5, Seongbuk-gu, Seoul, Republic of Korea; 6 Department of Biotechnology, Yonsei University, 50 Yonsei-ro, Seodaemun-gu, Seoul, Republic of Korea; 7 Department of Medical Education, California Northstate University College of Medicine, 9700 W Taron Drive, Elk Grove, CA, USA

## Abstract

Amyloid-β (Aβ) plays a critical role as a biomarker in Alzheimer’s disease (AD) diagnosis. In addition to its diagnostic potential in the brain, recent studies have suggested that changes of Aβ level in the plasma can possibly indicate AD onset. In this study, we found that plasma Aβ(1–42) concentration increases with age, while the concentration of Aβ(1–42) in the cerebrospinal fluid (CSF) decreases in APP_swe_, PS1_M146V_ and Tau_P301L_ transgenic (3xTg-AD) mice, if measurements were made before formation of ThS-positive plaques in the brain. Our data suggests that there is an inverse correlations between the plasma and CSF Aβ(1–42) levels until plaques form in transgenic mice’s brains and that the plasma Aβ concentration possesses the diagnostic potential as a biomarker for diagnosis of early AD stages.

The diagnostic potential of plasma amyloid-β (Aβ) in Alzheimer’s disease (AD) has been receiving attention because previous clinical studies indicated a possible relationship between increased risk of AD and lowered Aβ(1–42)/Aβ(1–40) ratio due to the decrease in plasma Aβ(1–42) concentrations^1^,^2^,^3^,^4^,^5^. A meta-analysis of 13 studies that assessed the potential of plasma Aβ as a diagnostic tool also reported a possibility of increased plasma Aβ(1–40) leading to subsequent cognitive decline^6^. If the Aβ concentration changes in the plasma can reflect the progression of AD in patients, it would enable the diagnosis of AD using less costly and less invasive methods. However, because there are studies that found either decreased or no meaningful changes in the plasma Aβ level of AD patients^7^,^8^,^9^, the potential of using the plasma Aβ measurements for AD diagnosis has been continuously debated. At this point, there is not enough evidence to acknowledge the level of plasma Aβ as a reliable biomarker for AD diagnosis yet.

When human AD studies build their AD patient pool, the diagnosis of probable AD is made based on the patient’s cognitive impairments described by the criteria of the National Institute of Neurological Disorders and Stroke and the AD and Related Disorders Association^10^,^11^. These human studies select patients with clinical diagnosis and study their Aβ abnormalities because the Aβ plaque deposition proceeds cognitive impairments in AD patients^12^,^13^. Current AD diagnosis can be confirmed with Aβ abnormalities detected by neuroimaging. Alternatively, measurements of Aβ in the cerebrospinal fluid (CSF) is known to reflect AD pathology in the brain^14^,^15^,^16^. If there were a method to diagnose AD prior to the Aβ plaque deposition in the brain, diagnosis and treatment of patients in early AD stages would be possible, even before at risk individuals develop cognitive impairments. This possibility led us to search for plasma biomarkers with diagnostic potentials for early AD stage.

A significant correlation between Aβ concentrations in the CSF and plasma were identified when we measured the Aβ levels prior to the plaque formation in the brain of transgenic mice. However, such correlations gradually disappeared as our mice aged and developed Aβ plaques in their brains. We hypothesized that the concentration changes in plasma Aβ can be utilized as a reliable biomarker for early diagnosis of AD prior to the plaque formation. In order to evaluate our hypothesis, we selected APP_swe_, PS1_M146V_ and Tau_P301L_ transgenic (3xTg-AD) mice, which imitate human AD pathophysiologies including age-dependent behavioral and cognitive alterations. The 3xTg-AD mice show cognitive deficits in the Morris water maze at 6 months of age and present visible Aβ plaque deposition in the brain at 6 months of age^17^,^18^,^19^,^20^,^21^. They are also known to develop Aβ plaques more slowly compared with other transgenic mice, making the 3xTg-AD model fit for studying the relationship between AD and the plasma and CSF Aβ level alterations before the plaque formation.

In this study, we first confirmed the absence of ThS positive amyloid plaques, dense-core plaques, in the brains of young 3xTG-AD mice with immunohistochemical staining. We then measured the changes in concentration of Aβ(1–42) and Aβ(1–40) in the CSF and plasma of 5-, 7-, 9- and 12-month-old female transgenic mice using sandwich-ELISA to define a relationship between the plasma Aβ and AD progression and, also, to confirm the diagnostic potential of the plasma Aβ prior to the plaque formation. We previously found that soluble monomeric Aβ(1–42) in the brain could pass the blood-brain barrier and can be found in the plasma^22^. To further confirm our hypothesis regarding the impact of plaque formation, we also assessed the permeability of the blood-brain barrier to insoluble fibrillary Aβ(1–42) in 7-week-old ICR mice and studied whether plasma Aβ(1–42) can reflect the condition of brain Aβ(1–42) even after the plaque deposition.

## Results

### Age-dependent accumulation of Aβ plaques in 3xTg-AD mouse brains

**Figure 1:**
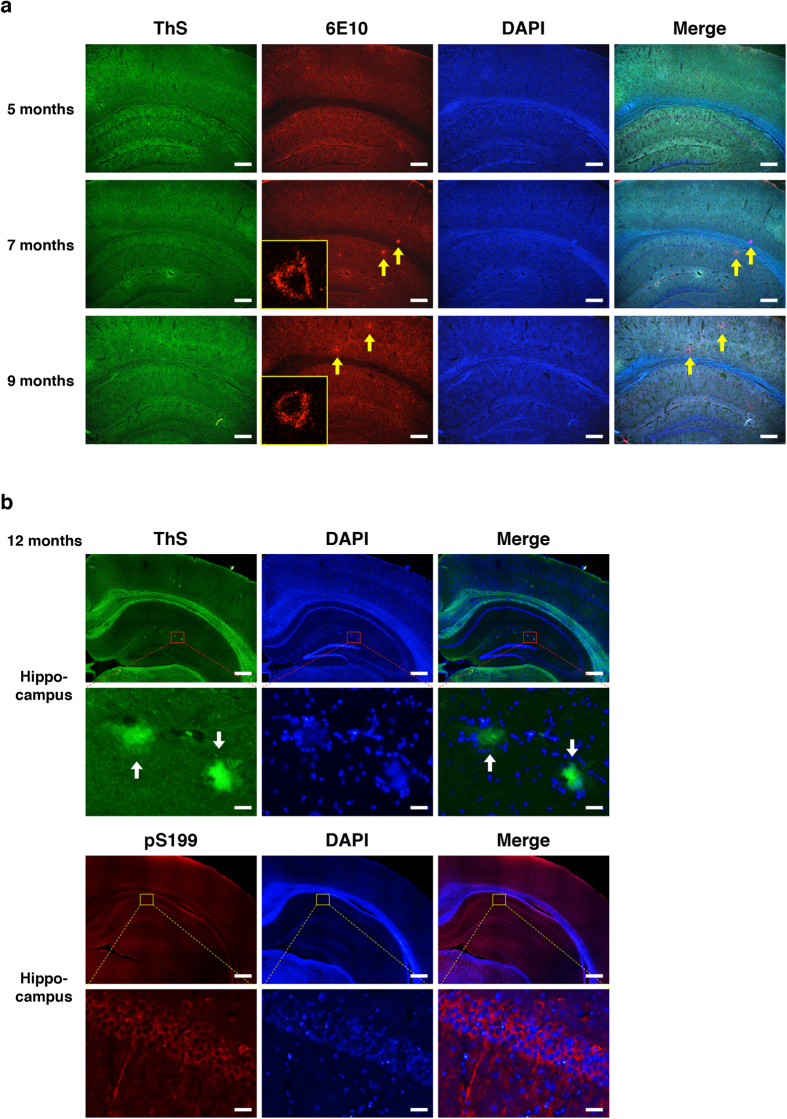
Age-dependent Aβ plaque deposition and hyperphosphorylated tau in the 3xTg-AD mice brain stained by ThS and IHC staining. 5-, 7-, 9- and 12-month-old female 3xTg-AD AD mice brains stained with ThS for β-sheet-rich Aβ plaques, 6E10 for detection of Aβ species and pS199 for hyperphosphorylated tau. (**a**) Hippocampus and cortex of 5-, 7-, 9- and 12-month-old female 3xTg-AD mice stained for Aβ plaques. Yellow boxes indicate whole-cell 6E10 immunostaining by confocal microscopy. Scale bar = 200 μm. (**b**) Hippocampus of 12-month-old female 3xTg-AD mice stained for hyperphosphorylated tau and Aβ plaque. Scale bar = 1 mm (*upper*) and 200 μm (*lower*). Yellow arrows indicate diffused Aβ plaque and white arrows indicate ThS positive Aβ plaque.

Here, female 3xTg-AD mice aged 5, 7, 9 and 12 months were selected to study changes in the plasma and CSF Aβ levels since ThS-positive Aβ plaques become detectable in the brain as early as 12 months of age (female; 5-month, n = 32; 7-, 9- and 12-month, n = 33)^20^. To confirm the absence of ThS-positive Aβ plaque in the brains of transgenic mice prior to Aβ measurements, brain samples from each age group were cyrosectioned and fixed with 4% paraformaldehyde for 72 hours, which is different from widely used protocols in other studies^21^,^23^. Then, they were stained with ThS for β-sheet-rich Aβ dense-core plaques, 6E10 antibody for both Aβ diffuse and dense-core plaques, DAPI for overall brain visualization and pS199 for hyperphosphorylated tau. We did not observe neither ThS- nor 6E10-positive plaques in the brain of 5-month-old mice (Fig. 1a). 6E10 immunostaining showed diffuse plaques in the hippocampal regions of 7-, 9- and 12-month-old female 3xTg-AD mice (Fig. 1a,b). In addition to the extracellular 6E10-positive plaques, we observed 6E10-stained neural cells from 7-month-old 3xTg-AD mice (Fig. 1a). Of the ThS stained brain slices, only the 12 month-old 3xTg-AD mice showed ThS-positive, dense-core, Aβ plaque depositions in the brain (Fig. 1b). In addition, we observed accumulations of hyperphosphorylated tau tangles in the brain of 12-month-old 3xTg-AD mice.

### Age-dependent Aβ(1–42) concentration changes in the CSF of 3xTg-AD mice

**Figure 2:**
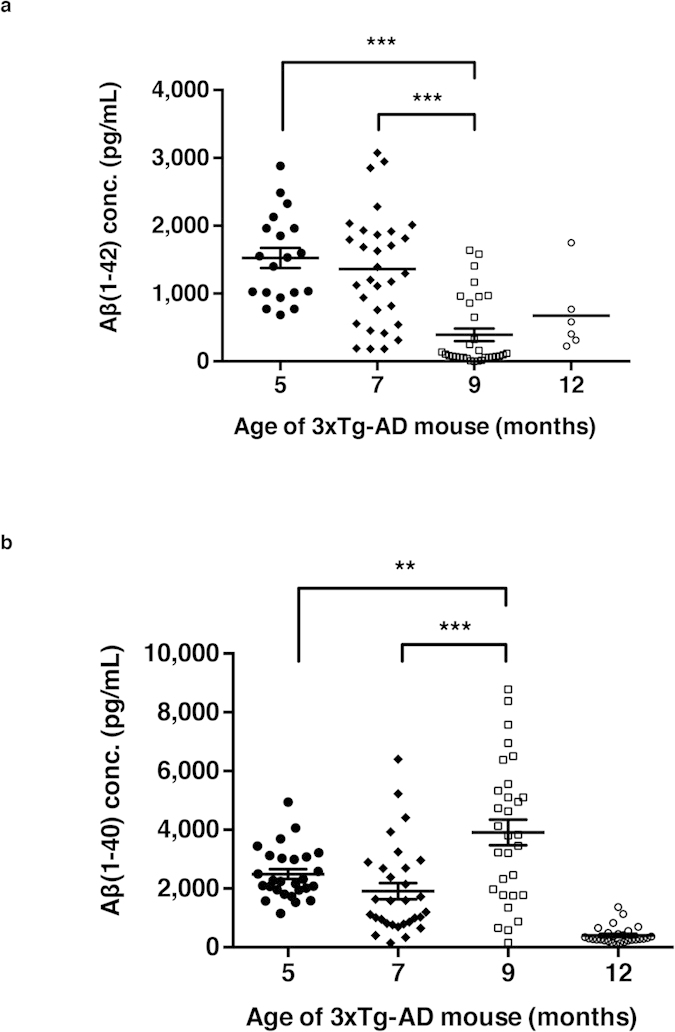
Age-dependent changes in Aβ(1–42) and Aβ(1–40) concentrations in the 3xTg-AD mice CSF (excluding saturated data). 5-, 7-, 9- and 12-month-old female 3xTg-AD AD mice Aβ in the CSF were measured using sandwich-ELISA. Data presented with scatter dot plot (mean ± SEM). (**a**) Aβ(1–42) concentration in CSF (5-month, n = 19; 7-month, n = 31; 9-month, n = 31; 12-month, n = 6) and (**b**) Aβ(1–40) concentration in CSF (5-month, n = 27; 7-month, n = 30; 9-month, n = 30; 12-month, n = 30). The error bars represent the SEMs. One-way ANOVA followed by Bonferroni’s post-hoc comparisons tests were performed in all statistical analyses (*P < 0.05, **P < 0.01, ***P < 0.001; other comparisons were not significant).

Decreased Aβ levels in CSF is a clinical indication of AD progression. To confirm the alterations of CSF Aβ levels in 3xTg-AD mice mimics those in AD patients, the levels of Aβ(1–42) in the CSF of 5-, 7-, 9- and 12-month-old 3xTg-AD mice were measured with sandwich-ELISA utilizing two anti-Aβ antibodies with different epitopes. The CSF was collected using laboratory-produced capillary tubes with tapered tips as previously described^24^ and then we measured the levels of Aβ(1–42) and Aβ(1–40). The concentration of Aβ(1–42) in the CSF of 3xTg-AD mice showed an age-dependent decrease with a statistical significance before the age of 12 months (female; 5-month, n = 19; 7-month, n = 31; 9-month, n = 31; 12-month, n = 6, *P* < 0.0001 for 5-month-old *vs*. 7-month-old and 7-month-old *vs*. 9-month-old, Fig. 2a)(13 out of 32 brains from 5-month-old and 27 out of 33 brains from 12-month-old groups were excluded from the results due to saturated values during ELISA readings). After the formation of ThS-positive plaques, the CSF Aβ(1–42) level no longer showed a decrease in the trend. On the contrary, we did not observe distinguishable trend in the age-dependent alterations of CSF Aβ(1–40) levels (*P* = 0.0049 for 5-month-old *vs*. 9-month-old, *P* < 0.0001 for 7-month-old *vs*. 9-month-old, Fig. 2b). Our results agree with previous studies on the age-dependent decline of CSF Aβ(1–42) levels in both AD patients and animal models.

### Age-dependent Aβ(1–42) concentration changes in the plasma of 3xTg-AD mice

**Figure 3:**
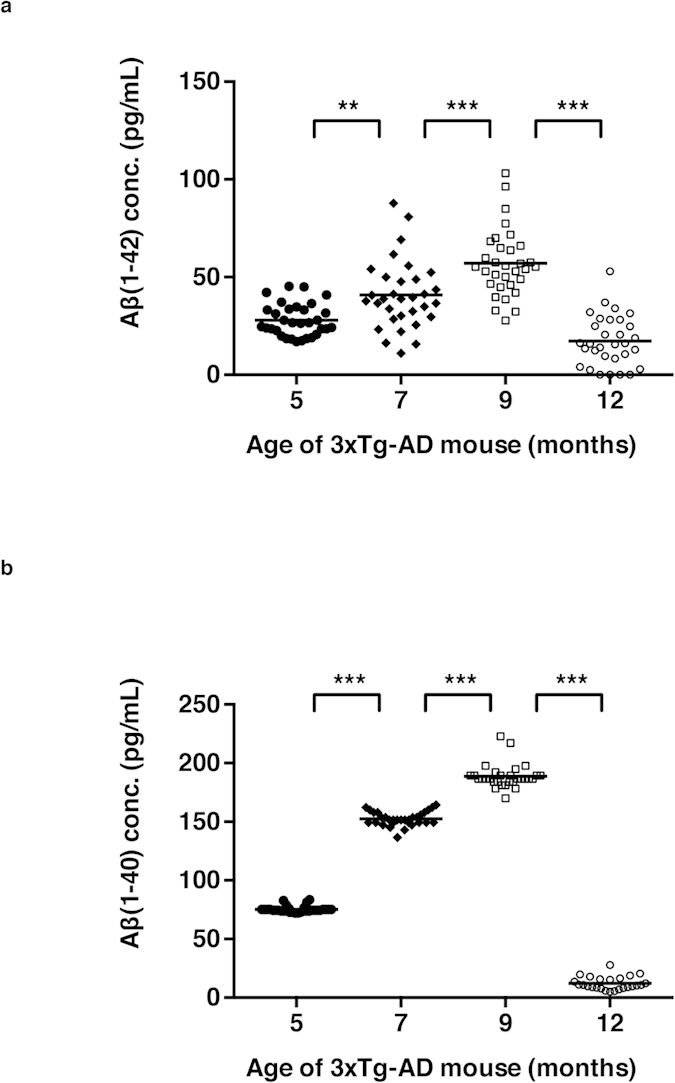
Age-dependent changes in Aβ(1–42) and Aβ(1–40) concentrations in the 3xTg-AD mice plasma. 5-, 7-, 9- and 12-month-old female 3xTg-AD mice Aβ(1–42) in the plasma were measured using sandwich-ELISA. Data presented with scatter dot plot (mean ± SEM). (**a**) Aβ(1–42) concentration in plasma (5-month, n = 32; 7-month, n = 32; 9-month, n = 32; 12-month, n = 31) and (**b**) Aβ(1–40) concentration in plasma (5-month, n = 32; 7-month, n = 31; 9-month, n = 30; 12-month, n = 25). The error bars represent the SEMs. One-way ANOVA followed by Bonferroni’s post-hoc comparisons tests were performed in all statistical analyses (*P < 0.05, **P < 0.01, ***P < 0.001; other comparisons were not significant).

In order to study if the plasma Aβ level changes in an age-dependent manner in the 3xTg-AD mice, we measured the concentration of plasma Aβ(1–42) in the aforementioned mice used in the CSF Aβ measurements. The blood was first transferred directly from the vena cava to EDTA-containing tubes and the plasma was isolated using centrifugation. Then we measured the levels of Aβ(1–42) and Aβ(1–40). In contrast to the CSF measurements, we found that plasma Aβ(1–42) levels increased from 5- to 9-month-old 3xTg-AD mice (female; 5-month, n = 32; 7-month, n = 32; 9-month, n = 32, Fig. 3a). Notably, the plasma Aβ(1–42) levels decrease in 12-month-old mice, of which brains only developed ThS-positive dense core plaques and hyperphosphoryated tau tangles (12-month, n = 31, Fig. 3a). All the results were statistically significant by one-way ANOVA followed by Bonferroni’s post-hoc comparisons (*P* = 0.003 for 5-month-old *vs*. 7-month-old, *P* < 0.0001 for 7-month-old *vs*. 9-month-old, *P* < 0.0001 for 9-month-old *vs*. 12-month-old). We observed identical trends in the alterations of plasma Aβ(1-40) to those of plasma Aβ(1–42). Between 5-, 7- and 9-month-old groups, 3xTg-AD mice showed significantly increasing levels of Aβ(1–40) in the plasma, whereas the 12-month-old group showed substantial decline (*P* < 0.0001 for 5-month-old *vs*. 9-month-old, *P* < 0.0001 for 7-month-old *vs*. 9-month-old, Fig. 3b).

**Figure 4:**
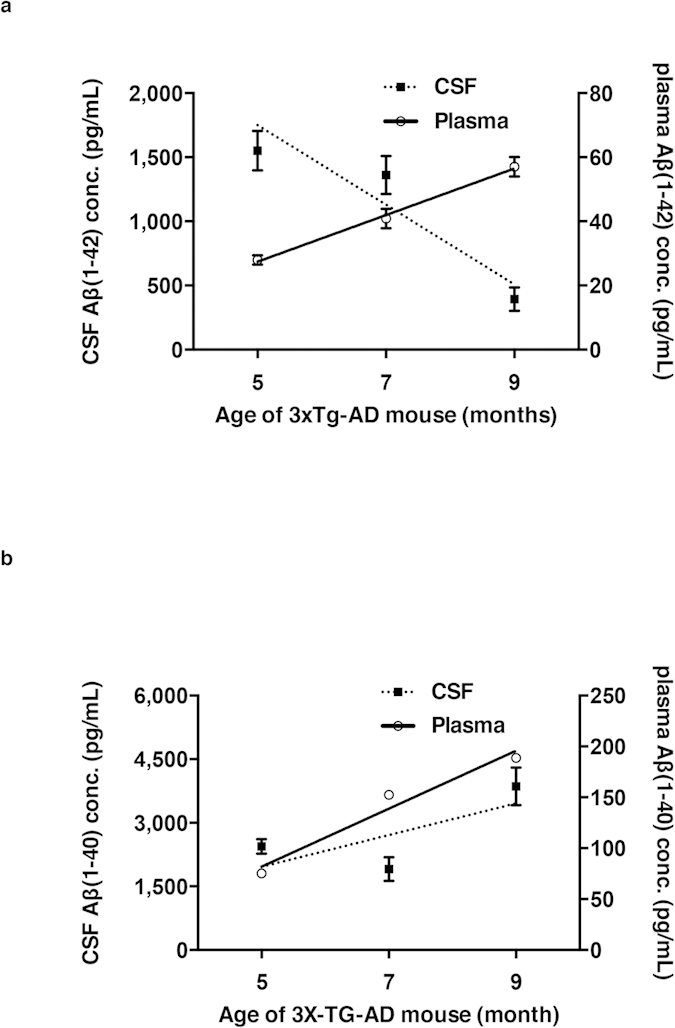
Comparison of age-dependent changes in Aβ(1–42) and Aβ(1–40) concentrations in 3xTg-AD mice between CSF and plasma. (**a**) Correlation of Aβ(1–42) concentrations between CSF and plasma of 5-, 7- and 9-month-old female 3xTG AD mice (CSF: *r* = −0.9324, *P* < 0.0001/Plasma: *r* = 0.9978, *P* < 0.0001). (**b**) Correlation of Aβ(1–40) concentrations between CSF and plasma of 5-, 7- and 9-month-old female 3xTG AD mice (CSF: *r* = 0.7030, *P* < 0.005/Plasma: *r* = 0.9791, *P* < 0.0001). The error bars represent the SEMs. One-way ANOVA followed by Bonferroni’s post-hoc comparisons tests were performed in all statistical analyses (*P < 0.05, **P < 0.01, ***P < 0.001; other comparisons were not significant).

To directly compare the age-dependent alterations in Aβ levels, the Aβ(1–42) and Aβ(1–40) concentrations in the plasma and CSF were plotted onto the same graph. The graph shows that the Aβ(1–42) concentrations in the plasma increases with age while the Aβ(1–42) concentrations in the CSF decreases, indicating inverse correlations between the Aβ(1–42) levels in the plasma and CSF (Fig. 4a). However, as there was no trend in CSF Aβ(1–40) levels, it was difficult to conclude any correlations between the Aβ(1–40) levels in the plasma and CSF (Fig. 4b). Collectively, our study concludes that the plasma Aβ(1–42) increases in age-dependent manner in 3xTg-AD mice and has the diagnostic potential for detecting AD until ThS-positive Aβ plaques form in the brain.

### Limited blood-brain barrier transport of fibrillary Aβ

**Figure 5:**
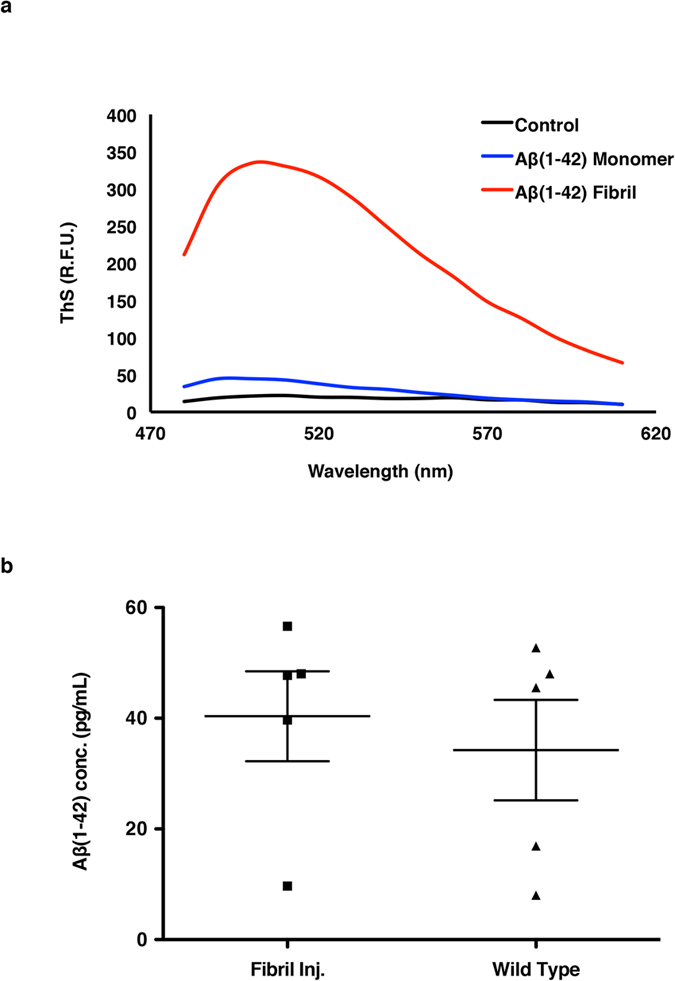
Evaluation of plasma Aβ(1–42) concentrations and ThS staining of fibirillary Aβ(1–42) after IC injection. (**a**) Excitation spectra of ThS (λex = 430 nm) in the presence of vehicle, Aβ(1–42) monomer and fibrillary Aβ(1–42). R.F.U. refers to relative fluorescence units. (**b**) Plasma Aβ(1–42) concentrations of 7-week-old male ICR mice that received IC injection with fibrillary Aβ(1–42) and control ICR mice that received vehicle were measured using sandwich-ELISA. Data presented with scatter dot plot (mean ± SD). The error bars represent the SEMs. Unpaired student’s *t*-tests were performed in all statistical analyses (the comparison was not significant).

Because the blood-brain barrier is known to be permeable to soluble monomeric Aβ(1–42), plasma Aβ(1–42) is a viable candidate for early AD diagnosis. However, if the changes in plasma Aβ(1–42) levels can serve as a biomarker for AD only prior to the plaque formation in the brain, then the plasma should not be able to reflect the condition of brain Aβ(1–42), once the Aβ peptides become insoluble aggregates. Direct fibrillary Aβ injection to tissue enabled us to study the blood-brain barrier’s permeability to insoluble Aβ by measuring the levels of fibrillary Aβ in the plasma. As we observed ThS-negative diffuse plaques did not have an effect on the plasma levels of Aβ in 3xTg-AD mice, it is important to inject ThS-postive Aβ aggregates. Thus, we performed ThS fluorescence assays to confirm formation of ThS-positive aggregates before the intracerebral (IC) injections (Fig. 5a). Then, we injected ThS-positive fibrillary Aβ(1–42) to the cortex of the brains of 7-week-old ICR mice (male, n = 5) by IC injection and assessed the permeability of the blood-brain barrier to fibrillary Aβ. We selected non-transgenic ICR mice for this study, since Aβ in the CSF of transgenic mice can potentially behave as a confounding factor. Additional 5 male ICR mice received IC injection with vehicle as controls. The blood was collected from the vena cava of the mice 30 minutes after the IC injection to allow sufficient time for fibrillary Aβ to pass the blood-brain barrier. After analyzing the plasma with sandwich-ELISA, no statistically significant differences in the plasma soluble Aβ concentrations of fibrillary Aβ-injected mice and vehicle-injected mice were found (Unpaired student’s *t*-test, *P* = 0.629, Fig. 5b). Therefore, the brain-blood barrier was not freely permeable to fibrillary Aβ(1–42), and the plasma Aβ(1–42) could not reflect the condition of brain Aβ(1–42) once they become insoluble plaques.

## Discussion

In this study, we found that the plasma Aβ(1–42) concentration increases in an age-dependent manner, while the level of CSF Aβ(1–42) decreases, indicating an inverse correlation between the plasma and CSF Aβ(1–42) levels in 3xTg-AD mice before dense-core Aβ plaque depositions appear in their brains. On the contrary, we did not observe such correlation in the alterations of Aβ(1-40) concentration between CSF and plasma. These results suggest that measuring the plasma Aβ(1–42) levels can function as an early diagnostic marker of AD. One previous comparison study involving the APP23 transgenic mice and the TG2576 mice also reported increasing plasma Aβ concentrations in age-dependent manner^25^, and another comparison study reported that in the amyloid precursor protein transgenic mouse models, Aβ concentrations in CSF decreases when Aβ deposition starts to appear^15^, further supporting our inverse correlation of Aβ levels. Although other additional studies have investigated Aβ levels in the CNS, they did not directly compare the Aβ levels in the CSF and plasma before and after plaque formation. Our study has further determined the aforementioned issue.

Our results indicate that the plasma Aβ(1–42) concentration possesses a diagnostic potential as a biomarker for early diagnosis of AD when there is no ThS positive Aβ plaque depositions in the brain. This finding is important because it could provide a possible explanation for controversial results from previous studies on the Aβ measurements in blood. Although the plasma Aβ was thought to be related to AD progression and was suggested as a potential target for AD diagnosis, previous controversial results made it difficult to draw a meaningful conclusion. Our study shows that the plasma Aβ(1–42) levels increases with age and, therefore, it can be used as an early marker for AD progression. However, the plasma Aβ may not be an ideal biomarker for AD diagnosis in later developing stages of AD, since amyloid plaque formation makes the plasma Aβ measurements unreliable. The plasma Aβ(1–42) levels may become unable to reflect the AD prognosis after the plaque deposition because insoluble Aβ(1–42) cannot pass the blood-brain barrier as seen in this study. However, one study reported possible endothelial damages to the blood-brain barrier by Aβ(1–42) peptides, which will impair the barrier’s function and increase its permeability^26^. Therefore, further study is recommended to warrant the diagnostic potential of plasma Aβ(1–42) levels after the plaque deposition in the brain.

It is not clear yet how the formation of amyloid plaques in the brain affects the age-dependent correlations between the plasma and CSF Aβ levels that we observed. Moreover, we only observed such correlations in female transgenic mice, which are known to develop amyloid plaques earlier than males^27^,^28^. Thus, the underlying mechanism causing the inverse correlation between the plasma and CSF Aβ(1–42) levels needs to be further studied, and investigations with the Dominantly Inherited Alzheimer Network are desired for more clinical data^29^. Nonetheless, this study opens up more therapeutic strategies for AD patients and could potentially lead to development of more convenient and economic diagnosis of AD in its early stages.

## Methods

### Materials

Zoletil^®^ (Virbac, France) and Rompun^®^ (Bayer Pharma, Germany) were purchased from SMP animal medicine (Korea). Protease inhibitor cocktail was purchased from Roche Diagnostics (USA). EDTA treated BD vacutainer^®^ was purchased from Becton, Dickinson and Company (USA). Human Aβ42 Ultrasensitive and Human Aβ40 ELISA Kit was purchased from Invitrogen (USA).

### Animals

B6;129-Psen1tm1Mpm Tg (APP_swe_, Tau_P301L_) 1Lfa/Mmjax mice (3xTg-AD) were obtained from Jackson Laboratory (USA) and then bred in a laboratory animal breeding room at the Korea Institute of Science and Technology. The mice were housed in groups of four per cage and maintained at constant temperature with an alternating 12-hour light-dark cycle. Food and water were available *ad libitum*. 129 mice were assessed in this study; 5-month-old 3xTg-AD (n = 32), 7-month-old 3xTg-AD (n = 33), 9-month-old 3xTg-AD (n = 33) and 12-month-old 3xTg-AD (n = 33). All animal experiments were performed in accordance with the National Institutes of Health guide for the care and use of laboratory animals (NIH Publications No. 8023, revised 1978). The animal studies were approved by the Institutional Animal Care and Use Committee of Korea Institute of Science and Technology (AP-2011L1015).

### CSF and plasma collection

CSF collection was performed according to the method described previously^24^. Mice were anesthetized with a blend of tiletamine.HCl, zolazepam.HCl (80 mg/kg, IP, Zoletil 50®, Virbac, France) and xylazine (20 mg/kg, IP, Rompun®, Bayer Pharma, Germany). The anesthetized mouse was placed horizontally, and its *cisterna magna* was surgically open. The opened meninges were stabbed with laboratory-produced capillary tube that had a tapered tip and achieved CSF. After CSF collection, the mouse was placed supinely, and its abdominal cavity was opened. Blood sampling from the vena cava was injected to EDTA tube with protease inhibitor cocktail (Roche Diagnostics, Switzerland, cat# 11836170001) and shaken gently. Plasma was separated from the blood. After centrifugation (3000 rpm, 15 minutes, 4 °C), CSF and plasma samples were stored at −80 °C freezer until use.

### ThS staining and immunohistochemistry

Animal were perfused with 0.9% NaCl, and the brains were fixed with 4% paraformaldehyde for 3 days and then were immersed in 30% sucrose solution for 3 days^23^,^30^. The brain samples were sliced at 35 μm using Cryostat (Microm HM 525, Thermo Scientific, USA) and mounted onto glass slides. Aβ plaques in cryo-sectioned brains were visualized by ThS staining. ThS was suspended in 50% of ethanol at 500 μM and brain sections were stained for 7 minutes. Then, to remove non-specific binding of ThS dye, the sections were rinsed through 100, 95 and 90% of ethanol for 10 seconds each and moved into PBS in succession.

To detect diffused plaque, brain slices were incubated in 0.3% PBS-T for permeabilization. After washing with PBS, brain slices underwent blocking step by 5% BSA for 1 hour and then incubated with primary antibody against Aβ (1:200, 6E10 clone, Covance, USA) overnight at 4 °C. Next day, brain slices were stained with Cy3 conjugated secondary antibody (1:400, Jackson ImmunoResearch, USA). To detect endogenous tau phosphorylation, brain slices were stained with primary antibody against pSer199 (1:200, Abcam, UK). Alexa fluor 633 congugated secondary antibody (1:500, Abcam, UK) was used for fluorocente detection. All slices were stained with Hoechst for counter staining. The images were taken with Leica DM2500 fluorescence microscope and the Cari Zeiss LSM700 confocal microscope.

### Aβ(1–42) and Aβ(1–40) analyzed by sandwich-ELISA in mice CSF and plasma

Aβ(1–42) levels in the CSF and plasma were quantified by Aβ42-ultra-sandwich-ELISA kit (Invitrogen, USA, cat# KHB3544) and Aβ(1–40) levels in the CSF and plasma were quantified by Aβ40-Human-ELISA kit (Invitrogen, USA, cat# KHB3482). The procedure was performed according to the manufacturer’s instructions. For the measurement of both Aβ(1–42) and Aβ(1–40) in the plasma samples were diluted 10-fold respectively. To measure Aβ(1–42) and Aβ(1–40) levels in CSF, CSF samples were diluted 2000-fold for Aβ(1–42) and 60-fold for Aβ(1–40), respectively. For all sample dilutions during the analysis, *Standard Diluent Buffer* was used. Diluted CSF and plasma samples were pipetted into each kit that NH_2_-terminus of human Aβ specific monoclonal antibody coated wells, and co-incubated with each Aβ specific monoclonal antibody. The intensity of this color was directly proportional to the concentration of human Aβ present in the CSF and plasma. All procedures were performed according to the manufacturer’s instructions same as both ELISA kits.

### Intracerebral injection of fibrillary Aβ(1–42)

ICR mice were obtained from ORIENT BIO Incorporated (Korea). Fibrillary Aβ(1–42) were prepared from the laboratory-produced synthetic Aβ(1–42) with 10% dimethyl sulfoxide (DMSO) that allow aggregation for 2 weeks at 37 °C and then centrifuged at 15,000 rpm for 5 minutes. After centrifugation, we discarded the supernatant and dissociated the pellet in 10% DMSO as the initial volume. Five 7-week-old ICR mice were IC injected with 5.85 μg fibrillary Aβ(1–42) in 1.3 μL according to previously published procedures^31^,^32^. 30 minutes after the IC injection, blood samples from the vena cava were collected and the plasma was separated from the blood. Soluble Aβ(1–42) levels in the plasma were quantified by Aβ42-ultra-sandwich-ELISA kit (Invitrogen, cat# KHB3544). The procedure was performed according to the manufacturer’s instructions as described previously.

### Fibrillary Aβ(1–42) detection

Quantitative aggregation of fibrillary Aβ(1–42) was monitored by ThS fluorescence assay. Mixtures of aggregated fibrillary Aβ(1–42) in PBS containing 2 μM ThS (Sigma, USA) were transferred to a black 384-well plate. ThS signal was measured in a Flexstation3 spectrophotometer (Molecular Devices, USA) with an excitation wavelength of 430  nm.

### Statistical analysis

Graphs were obtained with GraphPad Prism 5 and statistical analyses were performed with one-way ANOVA followed by Bonferroni’s post-hoc comparisons and Student’s *t*-test (*P < 0.05, **P < 0.01, ***P < 0.001; other comparisons were not significant). The error bars represent the SEMs. Results from statistical analyses are included in the (supporting information Table S1, S2).

## Additional Information

**How to cite this article**: Cho, S. M. *et al*. Age-dependent inverse correlations in CSF and plasma amyloid-β(1–42) concentrations prior to amyloid plaque deposition in the brain of 3xTg-AD mice. *Sci. Rep*. **6**, 20185; doi: 10.1038/srep20185 (2016).

## Supplementary Material

Supplementary Information
