# Supplementary material for: Age-dependent inverse correlations in CSF and plasma amyloid-β(1–42) concentrations prior to amyloid plaque deposition in the brain of 3xTg-AD mice
Source: Sci Rep. 2016 Feb 2;6:20185. doi: 10.1038/srep20185 (PMC4735736; doi:10.1038/srep20185)
Supplement: Supplementary Information [file srep20185-s1.pdf]

Supplementary Information

Correspondence and requests for materials should be addressed to Y.K. (yskim@bio.kist.re.kr)

Age-dependent inverse correlations in CSF and plasma amyloid-β(1-42) concentrations prior to amyloid plaque deposition in the brain of 3xTg-AD mice

Soo Min Cho, Sejin Lee, Seung-Hoon Yang, Hye Yun Kim, Michael Jisoo Lee, Hyunjin Vincent Kim, Jiyoon Kim, Seungyeop Baek, Jin Yun, Dohee Kim, Yun Kyung Kim, Yakdol Cho, Jiwan Woo, Tae Song Kim and YoungSoo Kim

Table S1. Statistical analyses of Aβ levels in the CSF (one-way ANOVA followed by Bonferroni's post-hoc comparisons).

Result from statistical analysis of plasma
One-way ANOVA
$F(3, 123) = 44.693$, $P = 0.000$
Bonferroni's post hoc analysis:
5 months vs. 7 months, $P = 0.003$
5 months vs. 9 months, $P = 0.000$
5 months vs. 12 months, $P = 0.024$
7 months vs. 9 months, $P = 0.000$
7 months vs. 12 months, $P = 0.000$
9 months vs. 12 months, $P = 0.000$
Other comparisons are not significant.

Table S2. Statistical analyses of Aβ levels in the plasma (one-way ANOVA followed by Bonferroni's post-hoc comparisons).

Result from statistical analysis of CSF
One-way ANOVA
$F(3, 83) = 15.638$, $P = 0.000$
Bonferroni's post hoc analysis:
5 months vs. 9 months, $P = 0.000$
7 months vs. 9 months, $P = 0.000$
Other comparisons are not significant.
